# Suspended Graphene-Based Gas Sensor with 1-mW Energy Consumption

**DOI:** 10.3390/mi8020044

**Published:** 2017-02-01

**Authors:** Jong-Hyun Kim, Qin Zhou, Jiyoung Chang

**Affiliations:** 1 Department of Mechanical Engineering; University of Utah, Salt Lake City, UT 84112, USA; jonghyun.kim@utah.edu; 2 Department of Mechanical Engineering; University of Nebraska–Lincoln, Lincoln, NE 68588, USA; zhou@unl.edu

**Keywords:** graphene, sensor, heater, NH_3_, oscillation, pulse, sensitivity, recovery, energy, flexible

## Abstract

This paper presents NH_3_ sensing with ultra-low energy consumption for fast recovery and a graphene sheet based on a suspended microheater. Sensitivity and repeatability are important characteristics of functional gas sensors embedded in mobile devices. Moreover, low energy consumption is an essential requirement in flexible and stretchable mobile electronics due to their small dimension and fluctuating resistivity during mechanical behavior. In this paper, we introduce a graphene-based ultra-low power gas detection device with integration of a suspended silicon heater. Dramatic power reduction is enabled by a duty cycle while not sacrificing sensitivity. The new oscillation method of heating improves the sensitivity of 0.049 (Δ*R*/*R*_0_) measured at a flow rate of 18.8 sccm NH_3(g)_ for 70 s. Our experimental tests show that a 60% duty cycle does not sacrifice sensitivity or recovery by dropping the total power consumption from 1.76 mW to 1.05 mW. The aforementioned low energy consuming gas sensor platform not only attracts environmentally-related industries, but also has the potential to be applied to flexible and stretchable mobile electronic devices.

## 1. Introduction

Graphene’s unique and excellent electrical and mechanical properties make it a tremendous contribution to flexible and stretchable electronics [1,2,3]. In particular, gas detection at low temperatures has been one of the main topics explored in applications based on 2D materials [4,5,6]. Among many gases, ammonia (NH_3_) is a one of major target compounds of toxic gases in the field of safety monitoring [2] Conventional toxic gas detection relies on catalytic reactions based on metal oxide, however recent study suggests that the sensitivity can be greatly enhanced by integration of atomically thin layered materials. There are multiple known advantages of graphene. First of all, graphene sheets can be used electronically in single electronic detectors operating at room temperature and in ultra-high sensitivity sensors with mechanical strain or magnetic fields [3]. In terms of mechanical aspects, graphene strength is an essential characteristic for wearable and embedded gas sensors in mobile devices, and graphene-based gas sensors are known to tolerate extreme sensitivity [4]. Thus, many attempts have been made recently to increase the performance of gas detection using chemical doping, array structures and UV-light exposure. The large arrays of sensors can raise the sensitivity due to broadening of the active detecting area [4]. However, there is a limit in size because small size and high sensitivity are required in order to match modern electronic devices. Increased concentration of charge carrier in graphene induced by adsorbed gas molecules can be useful in highly sensitive sensors [5]. Metal oxide semiconductor and solid electrolyte sensors with a wide typical detection range at an operating temperature of a few hundred degrees Celsius are commercially available. Although these types of sensors are inexpensive and robust, they require high energy consumption and cannot be fabricated on flexible substrates [6]. Other research revealed that the UV light exposure shows much improved sensitivity [7], but there are critical limitations to the method, specifically with respect to its reliability, as the sensor eventually loses its performance due to UV exposure. This is because single-walled carbon nanotube, sensing probes, are gradually removed by the continuous UV light irradiation [8]. In addition, several researchers focused on reducing recovery time. According to the experiment by Schedin et al., adsorbates can be removed by annealing the device at 150 °C [3]. Additionally, another experiment showed that the low temperature in range of 50~150 °C also increasingly contributed to molecular desorption. Semantic et al. fabricated micro-scale suspended hot plate arrays and deposited metals onto the plate by post-chemical vapor deposition (CVD) method [9,10]. The post-CVD method for metal deposition can provide thermal shock effects on the device. In addition, the study also indicated that the manufacturing process of the suspended heater structure is a challenging task. As a separate study, Fowler et al. made a suspended nitride heater and deposit graphene dispersion using spin-coating [11]. However, those suspended heaters have large dimensions (100–500 μm^2^), that are three-fold larger compared to the device in this paper. UV exposure for a short time offered an alternative to thermal annealing, however use of UV exposure is not recommended in general due to possible corrosion of carbon on the sensing sheet [8]. The temperature-programmed desorption curve using the Monte Carlo simulation methods and force-field parameters gives predictable data to grasp the fast recovery rate [12]. Therefore, there are clear needs for a device which can detect toxic gases at low power consumption without compromising sensitivity and recovery time. In this paper, a silicon-based microheater and single layered graphene are implemented to enable ultra-low energy consumption. The suspended structured heater is fabricated by surface micromachining followed by backside wet etching. Then, the graphene is deposited via poly-methyl-methacrylate (PMMA) transferring method. Then the sensing probe, covered with graphene, is formed by e-beam lithography. We have demonstrated ultra-low power consumption of up to 1.05 mW by implementing several factors including various input vibration parameters such as air-suspended microheaters, voltage, duty cycle and power input frequency. Multiphysics simulations are used to predict the temperature of a silicon heater and the results are used when designing a suspended heater. 

## 2. Device and Methods

### 2.1. Structure

**Figure 1:**
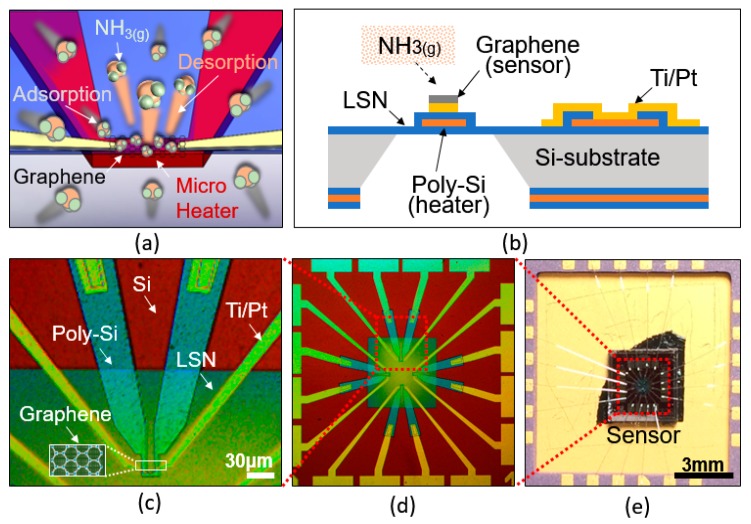
Schematic view of the sensor. (**a**) Concept of a microheater-based graphene gas sensor. Molecules are adsorbed on the graphene and fast recovery is achieved via heat-assisted desorbtion. (**b**) Cross-sectional view of the sensor. Heater is suspended in air to reduce heat loss. (**c**) The graphene is patterned on the bridge of the heater that generates high temperature due to narrow carrier pathway. (**d**) Top view of the device consists of four sensors on a single device. (**e**) A device is connected to a silicon-package by wirebonding. LSN: low-stress silicon-rich nitride.

Figure 1a shows the working principle of low-power gas sensor. Gas molecules are absorbed on the graphene and sensing electrodes placed on top of silicon nitride layer. A structural side view of the sensor is shown in Figure 1b. The microheater layer is sandwiched between two low-stress silicon nitride (LSN) membranes and suspended in the air to minimize heat loss. Ti and Pt are patterned to be electrically connected to the graphene and the packaging. A single layer of graphene on the copper film synthesized by CVD is transferred onto the device surface and patterned by e-beam lithography as shown in Figure 1c. The graphene sheet, as an absorber, is located on the narrow bridge region of the microheater layer. Each device has four sensors which perform independently and they are positioned on top, left, right and bottom in Figure 1d. Each sensor has four yellow-colored Ti/Pt legs at the center of the device. Two square shaped electrical connecting pads in the middle are opened to the input power source. The other two pads on the outside are connected to the electrical measurement setup. In Figure 1e, the device is mounted on a packaging platform that can be connected by wire bonding.

### 2.2. Working Principle

**Figure 2:**
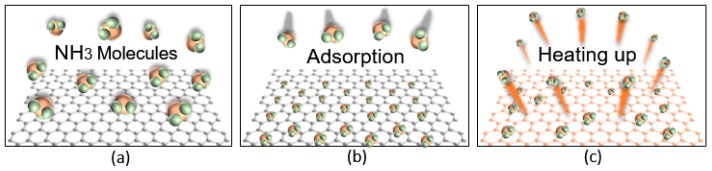
Conceptual process of interaction between molecules and graphene sheet. (**a**) The graphene has a steady condition without gas adsorption. (**b**) Molecules are adhered by interaction bonding. (**c**) Desorption occurs by air purging and annealing that accelerates desorption.

A mechanism of adsorption and desorption of NH_3_ molecules is shown in Figure 2. When the graphene surface is exposed to a stable environment composed of atmospheric pressure and room temperature, the graphene sheet exhibits an ohmic response before gas adsorption as shown in Figure 2a. When the graphene is exposed to NH_3_ as shown in Figure 2b, molecules are adhered on the graphene surface. This NH_3(g)_ sensing is based on changes in the resistivity due to molecular adsorption on the graphene sheet that act as donors [5]. Figure 2c shows desorption by air purification and annealing to accelerate the desorption rate. Molecular desorption reduces the electrical resistance by removing molecules from the graphene surface. The device is then purged with dry air to return graphene to its original resistance value [13]. In addition, the ultraviolet irradiation on graphene is known to increase the electrical resistance of the graphene [14]. Ultraviolet radiation catalyzes the sensitivity of graphene for sensing different types of gases, but graphene has weak light absorption and therefore light irradiation systems are difficult to apply to microelectronics [15]. The sensor’s sensitivity has a strong relationship with the activation temperature, therefore, accurately knowing the temperature is important.

**Figure 3:**
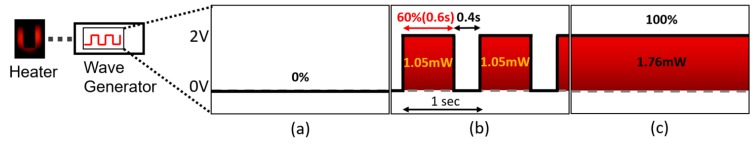
Schematic view of connection with microheater and power wave generator. (**a**) A 0% duty cycle means non-heating. (**b**) A 60% duty cycle of oscillation with 2 V shows 1.05-mW energy consumption. (**c**) Full duty cycle (continuous heating) generates 1.76 mW of energy consumption.

However, due to the size of the microheater, it is difficult to directly measure the temperature of the microheater. We have predicted the temperature of the heater to be about 200 °C by the 2 V input source using commercial finite element analysis (FEA) packaging. In addition, a silicon heater with the same dimensions was fabricated and tested with thermal couple on top of it to monitor the temperature variation along with voltage input. The heater’s micro-scale bridge can be used over a wide temperature range of up to 800 °C, driven by input power. The emission of a ‘U’ shaped microheater (bridge structure) on the left side of Figure 3 shows a silicon heater that glows around an input voltage of 8 volts with an expected temperature of 900 °C.

The zero duty cycle in Figure 3a has no energy consumption, and the 60% duty cycle with 2 V in Figure 3b means the generator releases 2 V power source for 0.6 s, then takes a rest for 0.4 s. Overall energy consumption during 1 s is equivalent to 1.05 mW of power consumption. In Figure 3c, the overall duty cycle shows a continuous power supply, reaching a higher energy consumption of 1.76 mW. Consolidating the duty cycle in the operation of the microheater greatly reduces power consumption.

### 2.3. Device Fabrication

**Figure 4:**
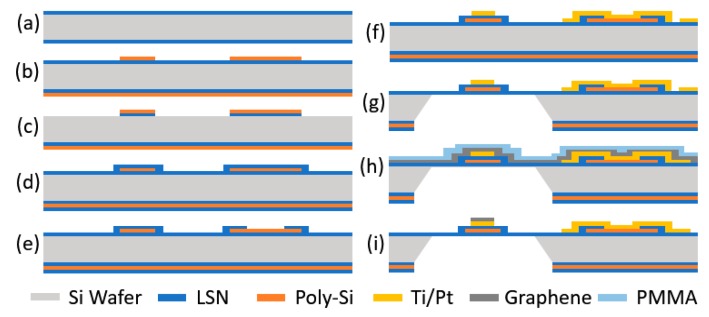
Fabrication process of suspended graphene gas sensor mounted on silicon based heater. Details of each process (**a**~**i**) are explained in above paragraph.

The fabrication process of the sensor is shown in Figure 4. (a) First, a low-stress silicon-rich nitride (LSN) of 100 nm is deposited on 500-µm-thick silicon substrate using low-pressure chemical vapor deposition (LP-CVD). (b) A boron-doped poly-silicon is deposited by CVD. Then the substrate is annealed at 1050 °C. Micro heater structure is patterned by photolithography process followed by reactive ion etching (RIE) of silicon. (c) LSN on the top surface is removed by plasma etching. (d) The LSN is deposited to make a sandwich structure on both sides of the heater. (e) The LSN is patterned to open the electrical connection between the poly-silicon and Ti/Pt that is formed. (f) Ti/Pt, 10/90 nm, are deposited on the top surface and patterned for the electrical pathway using supplemental items including a graphene connector and wire bonding pads. The device is annealed at 350 °C for 1 h in a nitrogen environment. (g) The silicon substrate is etched from the bottom by KOH solution for the suspended microheater. (h) The PMMA-coated graphene sheet is transferred onto the top surface. (i) The PMMA is patterned to shape the graphene sheet as an absorber on the heater region using e-beam lithography. The graphene layer is etched to define the heater region. Single-layer graphene synthesis is as follows.

The single-layered graphene is grown using the CVD process. A copper film is used for the growth substrate. The film is exposed to H_2_ for 100 sccm for 1 min and CH_4_ for 30 sccm for 3 min in the 550 °C furnace. Then, 10 sccm of H_2_ flows for 100 min in the 1050 °C furnace. After a pre-heating process, the film is exposed to both H_2_ of 10 sccm and CH_4_ 22 sccm for 85 min simultaneously. The furnace is shut-off after 25 min in a growth process. All gas valves are closed after 85 min. The film is stored in the furnace for cooling to room temperature overnight. Based on the characterization by Raman spectroscopy, the grown film is identified by matching the characteristic wavelength of the single layer graphene [16]. 

### 2.4. Measurement

**Figure 5:**
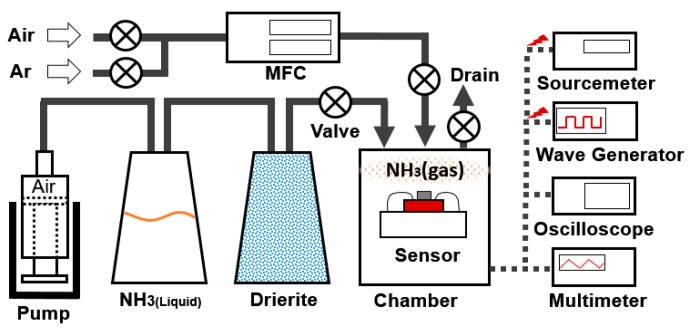
Experimental setup to flow target gas and measure the output from the gas sensor. MFC: mass flow controller.

Figure 5 shows an experimental setup for the gas adsorption and desorption. The test is performed in ambient pressure and room temperature environment. Here, 18.8 sccm of air is supplied from the syringe on the left side of the schematic image. The liquid form of ammonium hydroxide NH_3(L)_ evaporates and dissolves in the air. Then the air containing NH_3_ molecules is pumped into the next flask containing drierites, which absorbs water vapor from a mixture of NH_3_ and air. Then dry NH_3(g)_ molecules penetrate a test chamber. For desorption of the molecules, a wave form generator (SDG1025, Siglent, Shenzhen, China) is used to generate power for the heating. Multiple sets of temperatures and input power are tested by varying input voltage (0~2.5V), duty cycle (45%~100%) and frequency (1100 Hz). As a reference, the same set of tests is performed using argon_(g)_ gas. A multi-meter and a digital oscilloscope (TBS1052B, Tektronix, OR, USA) is used to measure the resistivity and wave form of the system. The detection capability is evaluated by the relative change in resistance per adsorption and desorption time (*R*_1_ − *R*_0_)/*R*_0_. NH_3__(g)_ 70-s adsorption and air desorption 70 s was repeated four times. This is a cycle that repeats every 70 s.

## 3. Results

**Figure 6:**
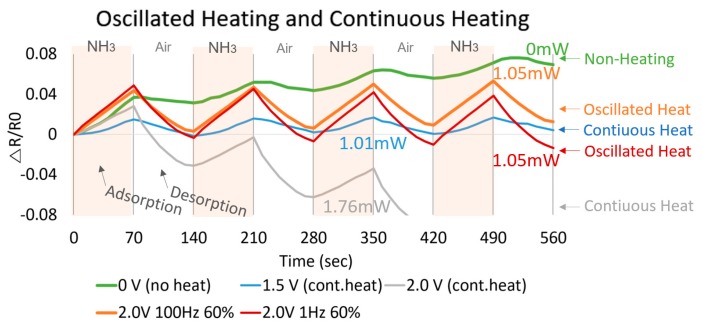
Comparison of sensing and recovery with varying duty cycles (0%, 60%, 100%). Adsorption cycle with NH_3(g)_ 18.8 sccm for 70 s and desorption cycle with air for 70 s are repeated for four cycles each. Ultra-low 1.05-mW energy consumption by optimized heating mode (2.0 V, 1 Hz, 60% duty cycle, red color) shows significant adsorption rate 0.049 (Δ*R*/*R*_0_) per 70 s of sensitivity and desorption rate 0.052 (Δ*R*/*R*_0_) per 70s of recovery.

**Figure 7:**
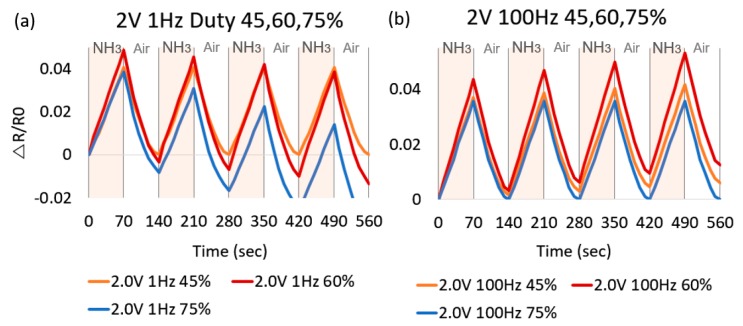
(**a**,**b**) shows a dependence of the frequency. A 60% Duty cycle achieves the highest 0.049 (Δ*R/R*_0_) of sensitivity. A 1-Hz frequency of all duty cycles shows higher sensitivity/recovery to gas. An optimized heating mode (2.0 V, 1 Hz, 60% duty cycle) shows the best performance.

The graphene-based sensor is evaluated as a change in electrical resistance based on NH_3_ gas and ultraviolet exposure. When the sensor is exposed to gas/UV, the resistance increases in both UV and NH_3_. However, low temperature heating between room temperature and 100 °C does not significantly improve sensitivity/recovery of UV detection. On the other hand, the adsorption sensitivity of NH_3_ increases by 40% when 0.5 V is applied compared to the same test conducted at room temperature. Argon (Ar) gas is supplied through a mass flow controller (MFC) in the gas tank. While heating from 100 to 200 °C, Δ*R*/*R*_0_ per 10 s recovery rate is monitored to be in the same range over all temperature ranges. The 200 sccm of Ar flow also provides 26% faster response than at 10 sccm. The air purging by uncovering with higher temperature gives a 6%~10% faster rate than 200 sccm Ar purging in detail. Using the air purging is the simplest method to refresh the sensor’s surface. In addition, the air is able to be regularly utilized when the sensor is operating on the wearable device. Thus, NH_3_ and the air worked in shifts for the evaluation of gas sensing and recovery. When the heater is heated to 100 °C or more by applying 1.5 V, the recovery speed is significantly reduced. In other words, the faster the recovery speed, the better the high voltage, but the constant power input of 2.0 V greatly reduces the sensitivity. Based on the tests, input power ranging between 1.5 and 2.0 V can serve as optimized refreshing input power, satisfying both reasonable recovery and sensitivity. As shown in Figure 6, increasing the temperature around 200 °C by 2 V input voltage with 60% duty cycle of oscillation improves the sensitivity and the recovery rate of NH_3(g)_ sensing. It is noted that a test without heating (no input power source) clearly shows the poorest recovery for the gas. Consolidating the duty cycle to reduce power consumption significantly reduces power consumption over continuous heating without compromising sensitivity and recovery time. If the total input power is fixed at 0.6 mW through 40% of the duty cycle, the influence of the input frequency is not apparent. A 1-Hz input frequency improves performance if the total input power is fixed at 1.76 mW over 75% of duty cycle and 1.05 mW over 60%. For example, red (1 Hz) represents a higher absorption and desorption response than orange (100 Hz) with a total input power of 1 mW. However, the 45% and 75% of duty cycles with 1 Hz of frequency give low adsorption/desorption rates that are 0.041/0.041 and 0.038/0.047 (Δ*R*/*R*_0_) per 70 s, respectively, compared to the rates 0.049/0.052 (Δ*R*/*R*_0_) per 70 s at 60%. Next, the NH_3(g)_ sensing device’s dependence on input power frequency is tested and a small noticeable difference is observed as shown in Figure 7a,b. The 60% duty cycle at 1 and 100 Hz reaches the highest Δ*R*/*R*_0_ of sensitivity. A 1-Hz frequency shows a higher sensitivity/recovery rate than 100 Hz. Lastly, in an optimal test condition, ultra-low 1.05-mW energy consumption is achieved by a 2.0-V 1-Hz 60% duty cycle input power source that shows as high as 0.049 (Δ*R*/*R*_0_) sensitivity with 18.8 sccm of NH_3(g)_ input flow.

## 4. Discussion

Various types of interaction, from weak van der Waals to strong covalent bonding, occur between adsorbed molecules and the graphene atoms during the experiment. This leads to a noticeable change in electrical conductivity of graphene [17]. Molecules adsorbed on the graphene sheet act as scattering centers, resulting in an increase in the resistivity of graphene [18]. Higher density of gas can decrease the greater conductivity. It can also be seen that, depending on the change in the emission of the microheater, the heater can generate various temperatures by changing the input power, as well as predicting the temperature in the low temperature range of 100 to 300 °C. The desorption is accelerated, especially with an input voltage of 1.5 V or more. This simulation shows that the 1.5 and 2.0 V input voltages produce approximately 100 and 200 °C respectively. Thus, the relationship between simulation and experimental data can accurately predict low heating temperatures. It is noted that when thermal energy is added, separation of molecules can be accelerated. This is because as the temperature increases, the hydrogen bond network is reorganized, and the NH_3_ molecules are gradually desorbed from the surface by breaking of their intermolecular hydrogen bonds [12]. Moreover, there are various binding energies between molecules and graphene within one hexagon of graphene [19]. Thus, the bindings are increasingly broken as time goes on when the graphene is heated. Heating generally increases the sensitivity and at the same time increases the desorption rate. However, when the continuous heating is applied, the sensitivity of continuous heating does not show significant improvements compared to both non-heating and optimized oscillations. The optimized oscillation input is therefore suitable for overall performance and energy consumption. To further reduce energy consumption, the sensor can employ an oscillating power delivery scheme that uses a variety of duty cycles, frequencies, and voltages. According to various sources, high temperature and continuous power can gradually increase the supported recovery rate by weakening the interatomic coupling due to absorption of external atomic energy.

## 5. Conclusions

Ultra-low power consumption with graphene and micro-electro-mechanical-system (MEMS) based gas sensors have been successfully fabricated and demonstrated. A suspended microheater structure plays a key role in reducing power consumption. In addition, the duty cycle is integrated to further reduce power consumption to 1.05 mW. Although NH_3_ gas is tested as one of the typical toxic gases, the platform is open to other types of gas detection through simple calibration. The sensor shows rapid recovery through heating of the silicon heater. As the temperature increases, the desorption gradually increases by reducing hydrogen bonding. With optimized configuration parameters, a 60% duty cycle of 2.0-V 1-Hz input power oscillation reduces energy consumption to 1.05 mW. The suspended microheater structure is suitable for preventing heat transfer which contributes to very low energy consumption. The pulsed input power may reduce damages of graphene and structures. The aforementioned low-energy consumption gas sensor platform will be an attractive platform for flexible and flexible mobile electronic devices.

## References

[1] Cho B., Yoon J., Hahm M.G., Kim D.H., Kim A.R., Kahng Y.H., Park S.W., Lee Y.J., Park S.G., Kwon J.D. (2014). Graphene-based gas sensor: metal decoration effect and application to a flexible device. J. Mater. Chem. C..

[2] Lee K., Scardaci V., Kim H.Y., Hallam T., Nolan H., Bolf B.E., Maltbie G.S., Abbott J.E., Duesberg G.S. (2013). Highly sensitive, transparent, and flexible gas sensors based on gold nanoparticle decorated carbon nanotubes. Sens.Actuators B Chem..

[3] Schedin F., Geim A.K., Morozov S.V., Hill E.W., Blake P., Katsnelson M.I., Novoselov K.S. (2007). Detection of individual gas molecules adsorbed on graphene. Nat. Mater..

[4] Sheehan P.E., Whitman L.J. (2005). Detection limits for nanoscale biosensors. Nano Lett..

[5] Leenaerts O., Partoens B., Peeters F.M. (2007). Adsorption of H_2_O, NH_3_, CO, NO_2_, and NO on graphene: A first-principles study.

[6] Inoue T., Ohtsuka K., Yoshida Y., Matsuura Y., Kajiyama Y. (1995). Metal oxide semiconductor NO_2_ sensor. Sens. Actuators B Chem..

[7] Mitoma N., Nouchi R., Tanigaki K. (2015). Enhanced sensing response of oxidized graphene formed by UV irradiation in water. Nanotechnology.

[8] Chen g., Paronyan T.M., Pigos E.M., Harutyunyan A.R. (2012). Enhanced gas sensing in pristine carbon nanotubes under continuous ultraviolet light illumination. Sci. Rep..

[9] Semancik S., Cavicchi R.E., Wheeler M.C., Tiffany J.E., Poirier G.E., Walton R.M., Suehle J.S., Panchapakesan B., DeVoe D.L. (2001). Microhotplate platforms for chemical sensor research. Sens. Actuators B Chem..

[10] Cavicchi R.E., Suehle J.S., Kreider K.G., Shomaker B.L., Small J.A., Gaitan M., Chaparala P. (1995). Growth of SnO_2_ films on micromachined hotplates. Appl. Phys. Lett..

[11] Fowler J.D., Allen M.J., Tung V.C., Yang Y., Kaner R.B., Weiller B.H. (2009). Practical chemical sensors from chemically derived graphene. ACS Nano..

[12] Liu L., Zhao L., Sun H. (2009). Simulation of NH_3_ temperature-programmed desorption curves using an ab initio force field. J. Phys. Chem. C..

[13] Zhang Z., Zhang X., Luo W., Yang H., He Y., Liu Y., Zhang X., Peng G. (2015). Study on adsorption and desorption of ammonia on graphene. Nanoscale Res. Lett..

[14] Akinwande D., Petrone N., Hone J. (2014). Two-dimensional flexible nanoelectronics. Nat. Commun..

[15] Novoselov K.S., Geim A.K., Morozov S.V., Jiang D., Zhang Y., Dubonos S.V., Grigorieva I.V., Firsov A.A. (2004). Electric field effect in atomically thin carbon films. Science.

[16] Liu Y., Liu Z., Lew W.S., Wang Q.J. (2013). Temperature dependence of the electrical transport properties in few-layer graphene interconnects. Nanoscale Res. Lett..

[17] Silvestrelli P.L. (2009). van der Waals interactions in density functional theory using wannier functions. J. Phys. Chem. A..

[18] Hwang E.H., Adam S., Das Sarma S. (2007). Transport in chemically doped graphene in the presence of adsorbed molecules. Phys. Rev. B.

[19] Lin X., Ni J., Fang C. (2013). Adsorption capacity of H_2_O, NH_3_, CO, and NO_2_ on the pristine graphene. J. Appl. Phys..

